# M1 macrophage dependent-p53 regulates the intracellular survival of mycobacteria

**DOI:** 10.1007/s10495-019-01578-0

**Published:** 2019-11-05

**Authors:** Yun-Ji Lim, Junghwan Lee, Ji-Ae Choi, Soo-Na Cho, Sang-Hun Son, Sun-Jung Kwon, Ji-Woong Son, Chang-Hwa Song

**Affiliations:** 1 Department of Microbiology, College of Medicine, Chungnam National University, Daejeon, 35015 South Korea; 2 Department of Medical Science, Chungnam National University, Daejeon, South Korea; 3 Research Institute for Medical Sciences, College of Medicine, Chungnam National University, Daejeon, South Korea; 4 Department of Internal Medicine, Konyang University Hospital, Daejeon, South Korea

**Keywords:** Apoptosis, Tuberculosis, Mycobacteria, Macrophages, p53, Polarization

## Abstract

Tumor suppressor p53 is not only affects immune responses but also contributes to antibacterial activity. However, its bactericidal function during mycobacterial infection remains unclear. In this study, we found that the p53-deficient macrophages failed to control *Mycobacterium tuberculosis* (Mtb), manifested as a lower apoptotic cell death rate and enhanced intracellular survival. The expression levels of p53 during Mtb infection were stronger in M1 macrophages than in M2 macrophages. The TLR2/JNK signaling pathway plays an essential role in the modulation of M1 macrophage polarization upon Mtb infection. It facilitates p53-mediated apoptosis through the production of reactive oxygen species, nitric oxide and inflammatory cytokines in Mtb-infected M1 macrophages. In addition, nutlin-3 effectively abrogated the intracellular survival of mycobacteria in both TB patients and healthy controls after H37Ra infection for 24 h, indicating that the enhancement of p53 production effectively suppressed the intracellular survival of Mtb in hosts. These results suggest that p53 can be a new therapeutic target for TB therapy.

**Electronic supplementary material:**

The online version of this article (10.1007/s10495-019-01578-0) contains supplementary material, which is available to authorized users.

## Introduction

Tuberculosis (TB) caused by *Mycobacterium tuberculosis* (Mtb) is estimated to affect more than one-fourth of the world’s population, and it is the most important bacterial infection worldwide. During Mtb infection, macrophages provide a critical first line of host defense [1]. Macrophages recognize Mtb antigens on the bacterial cell surface and secrete proteins in response to various receptors, including Toll-like receptors (TLRs), to modulate inflammatory responses and bactericidal functions [2]. Macrophages are classified as M1 and M2 according to their functions. Classically activated (M1) macrophages are polarized by lipopolysaccharide (LPS) and interferon γ (IFNγ), while alternatively activated (M2) macrophages are polarized by interleukin-4 (IL-4) and IL-13 [3, 4]. Generally, M1 macrophages produce pro-inflammatory cytokines, reactive oxygen species (ROS), and nitric oxide (NO), leading to bacterial death [5]. In a previous study, we showed that virulent mycobacterial infection skewed macrophages from the M1 to M2 type [6].

Apoptosis is an important mechanism in immune cells during bacterial and viral infection [7, 8]. Because many intracellular bacterial or viral pathogens evade the defenses of the immune system and hide within the host cell for their replication, the induction of apoptosis in infected cells can be used to limit their survival [9]. Recent studies have suggested that inducing the apoptosis of Mtb-infected host cells helps maintain host defense [10, 11]. Our previous studies have consistently indicated that apoptosis mediated by endoplasmic reticulum (ER) stress in macrophages benefits the host against Mtb infection [12–14]. Importantly, ER stress-mediated apoptosis effectively removes Mtb in M1-polarized macrophages more so than in M2 macrophages [6]. In this study, we hypothesized that M1-polarized macrophages might be useful for eliminating intracellular Mtb via the induction of pro-apoptotic-associated mechanisms such as p53-dependent apoptosis.

The tumor suppressor gene p53 is a transcription factor that promotes target genes associated with DNA repair, cell cycle arrest, senescence and programmed cell death, thereby limiting tumorigenesis [15, 16]. Upon phosphorylation and acetylation, activated p53 can directly bind to specific DNA sequences in the promoter regions of target genes including those regulating apoptosis, DNA repair, and the cell cycle [16–18]. p53 plays a key modulating role in the pro-apoptotic effect between the extrinsic and intrinsic pathways, through transcriptional regulation of its target genes such as p53 upregulated modulator of apoptosis (PUMA), NOXA, Bcl2-associated X (Bax) and BH3 interacting-domain death agonist (Bid), which release apoptotic proteins from the mitochondria, activating caspases and apoptosis [19].

The activation of p53 is initiated by oxidative stresses, including ROS and NO, which may in turn upregulate inflammation and programmed cell death. In addition, p53 promotes cytochrome c release and caspase activation, resulting in apoptotic cell death though mitochondrial ROS and NO generation. p53 interacts with the nuclear factor κB (NF-κB) [20] and mitogen-activated protein kinase (MAPK) pathways [21] in inflammatory and immune responses. Owing to these regulatory functions, we hypothesized that p53 is involved in the modulation of macrophage polarization. Recent evidences have revealed that the presence of p53 is important for infected cells to have a bactericidal effect in various infectious diseases, including influenza, pneumonia, chlamydia, listeriosis and *Helicobacter pylori* infections [22–27]. Mtb infection also increases p53 gene expression in a human monocytic cell line [28] and peripheral blood human monocytes [29]. A previous study showed that Mtb-induced tumor necrosis factor (TNF)-α modulates p53 expression in macrophages cell line [30]. However, the detailed functions of p53 during mycobacterial infection remain poorly understood.

In this study, we investigated the role of p53 in abrogating the intracellular survival of Mtb in macrophages. Our results revealed an antibacterial role of p53 through apoptotic cell death of M1-polarized macrophages during mycobacterial infection.

## Results

### The p53 expression in Mtb-infected macrophages controls intracellular survival

**Fig. 1:**
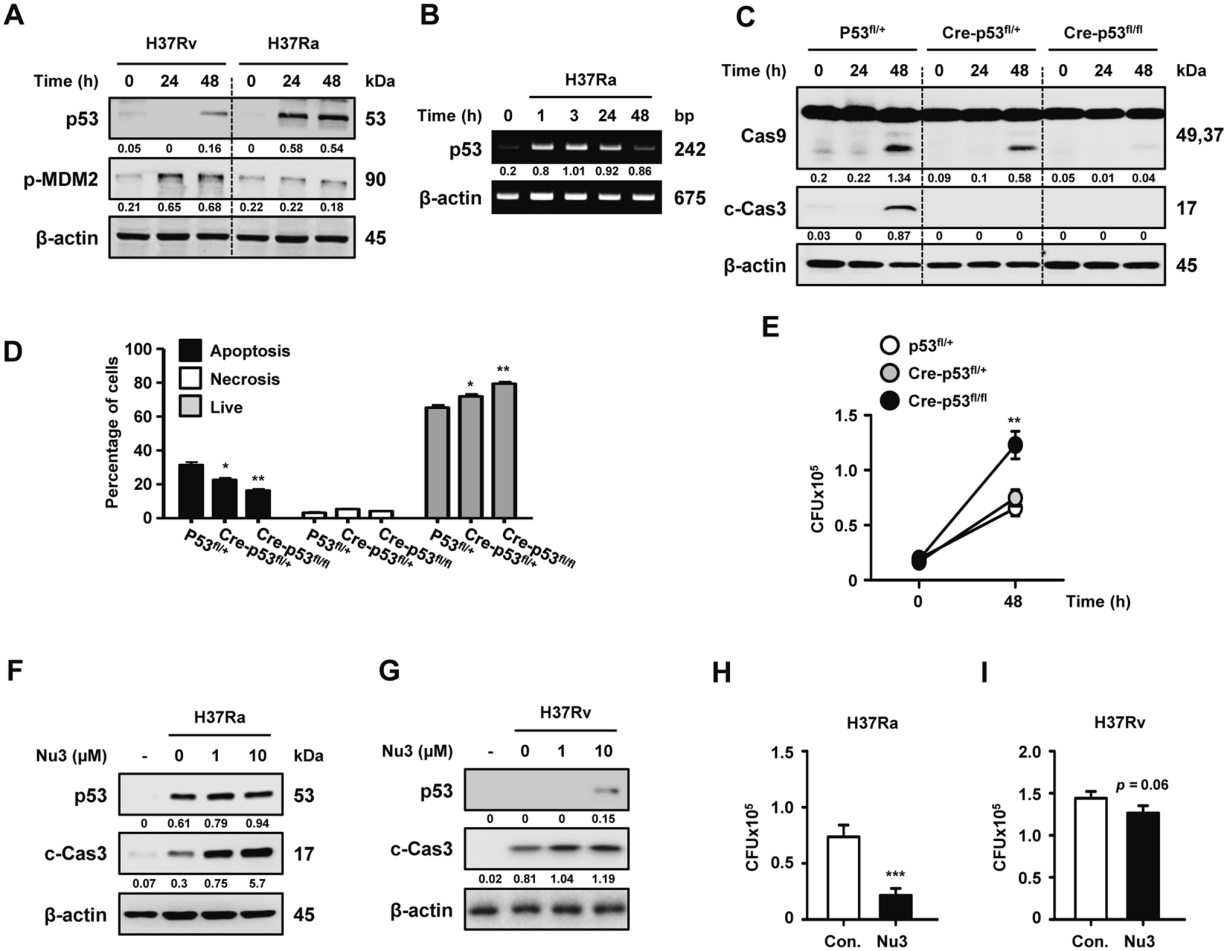
p53 expression in Mtb-infected macrophages causes apoptotic cell death to control the survival of intracellular mycobacteria. **a** BMDMs were infected with either H37Rv or H37Ra at a MOI of 1 for the indicated times. The expression levels of p53 and p-MDM2 proteins were analyzed using Western blotting. **b** H37Ra infection-dependent p53 mRNA expression was evaluated using reverse transcription–PCR in a time-dependent manner. **c** Activation of caspases 9 and 3, **d** apoptotic cell death, and **e** intracellular survival of Mtb after H37Ra infection for 48 h were measured in BMDMs from WT, Cre-p53^flox/+^, and Cre-p53^flox/flox^ mice. **f** H37Ra- and **g** H37Rv-infected macrophages were treated with nutlin-3 for 48 h, and p53 and caspase-3 activation were measured using Western blotting. Intracellular survival of **h** H37Ra and **i** H37Rv were determined in infected macrophages treated with nutlin-3 (10 μM). The results are representative of three independent experiments. Statistically significant differences are indicated; *statistically significant differences at a p-value < 0.05; **p-value < 0.01 versus WT or control groups

To determine whether p53 activation is increased in mycobacterial infection, we infected murine bone marrow-derived macrophages (BMDMs) with Mtb H37Rv (H37Rv) or Mtb H37Ra (H37Ra) and monitored the expression of p53 in a time-dependent manner. As shown in Fig. 1a, the production of p53 protein was significantly enhanced with Mtb infection. Attenuated H37Ra-infected macrophages produced higher levels of p53 production than virulent H37Rv, while H37Rv infection induced higher levels of mouse double minute 2 (MDM2) expression than H37Ra infection. Similarly, the mRNA levels of p53 were continually induced in H37Ra-infected BMDMs until 24 h post infection (Fig. 1b). Next, we checked p53-mediated apoptosis during Mtb infection using macrophages from p53 conditional knockout mice. LysM-Cre;p53^flox/flox^ mice showed deleting exons 2-10 of the p53 allele (Fig. S1a). p53-deleted macrophages from LysM-Cre;p53^flox/flox^ mice showed no production of p53 mRNA or p53 protein (Fig. S1b–c). Caspase-9/3 activation and apoptosis induction by Mtb infection were significantly suppressed in p53-deleted BMDMs from LysM-Cre;p53^flox/flox^ mice compared to those in wild-type (WT) mice, p53^flox/flox^, and p53^flox/+^ mice (Fig. 1c–d). In addition, the intracellular survival of Mtb was enhanced in p53-deleted macrophages than in p53 WT macrophages (Fig. 1e), which was followed by declined apoptotic cell death. By contrast, treatment with nutlin-3, a p53 activator that inhibits the p53–MDM2 interaction, dramatically increased the activation of p53 and caspase-3 during H37Ra infection (Fig. 1f). In H37Rv-infected BMDMs, p53 production and caspase-3 activation were induced by treatment with 10 μM nutlin-3. However, low dose of nutlin-3 (1 μM) didn’t induce p53 production (Fig. 1g). After 48 h of H37Ra infection, the intracellular survival rates of H37Ra in macrophages were remarkably repressed in the presence of nutlin-3 (approximately 29%) than in its absence (Fig. 1h). The intracellular survival of H37Rv tended to decrease in nutlin-3 treated macrophages (Fig. 1i). Taken together, our data suggest that the induction of p53-dependent apoptosis effectively reduces the intracellular growth of Mtb in macrophages.

### TLR2-MAPK signaling activation is essential for p53 activation in Mtb-infected macrophages

**Fig. 2:**
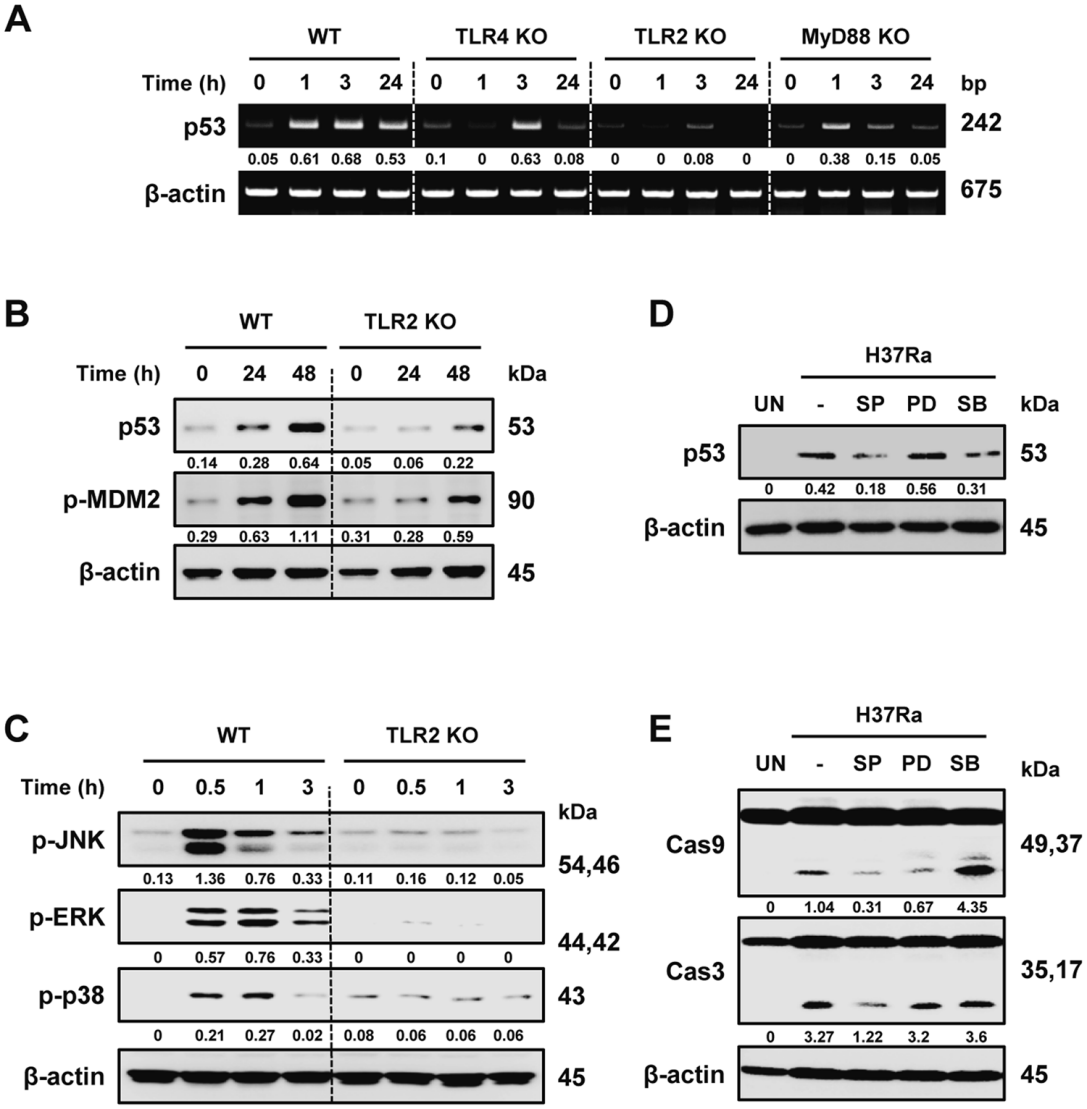
Activation of TLR2-dependent signaling in Mtb-infected macrophages is associated with p53 activation. **a** BMDMs from WT, TLR4-, TLR2-, and MyD88-deficient mice were infected with H37Ra and then analyzed for p53 mRNA levels. **b** The protein levels of p53 and p-MDM2 and **c** phosphorylation of JNK, ERK, and p38 were detected in WT and TLR2-deficient macrophages upon H37Ra infection. **d** BMDMs were pretreated with a specific inhibitor of JNK (SP600125, 30 μM), ERK (PD098059, 50 μM), or p38 (SB203580, 10 μM) for 2 h and then were infected with H37Ra for 48 h, followed by analyses of **d** p53 and **e** caspase activation

TLR pathway-triggered apoptosis is intimately associated with p53 signaling activation [31]. To investigate the role of the TLR signaling pathway in the production of p53 during Mtb infection, we studied TLR4-, TLR2-, and MyD88-deficient BMDMs. The induction of p53 mRNA expression was suppressed in TLR2- and MyD88-deficient BMDMs during H37Ra infection (Fig. 2a), and the production of p53 protein was also decreased in TLR2 KO macrophages than in WT cells (Fig. 2b). Simultaneously, p53 expression following H37Ra infection in TLR4-deficient BMDMs was similarly induced in WT cells (Fig. S2a). Next, we examined TLR2-activated MAPK signaling in Mtb-infected macrophages because it is a major downstream component of TLR signaling stimulation. The MAPK pathway, which is mediated by c-JUN N-terminal kinase (JNK), extracellular signal-regulated kinase (ERK), and p38, was triggered in H37Ra-infected WT macrophages to a higher level than in TLR2 KO macrophages (Fig. 2c). However, activation of JNK, ERK, and p38 caused by Mtb infection was not affected by p53 deficiency (Fig. S2b). In addition, H37Ra-induced p53 expression was suppressed in the presence of SP600125 (JNK inhibitor) and SB203580 (p38 inhibitors) but in the presence of PD98059 (ERK inhibitor) (Fig. 2d). Furthermore, the levels of p53 expression and activated caspases were suppressed in H37Ra-infected TLR2-deficient BMDMs (Fig. S2c). SP600125 treatment selectively repressed the activation of caspase-9 and caspase-3 during Mtb infection, while PD98059 and SB203580 did not have inhibitory effects (Fig. 2e). Sequentially, TLR2-deficient BMDMs failed to control intracellular Mtb replication as bacterial CFUs (Fig. S2d). Altogether, these findings suggest that JNK activation through TLR2 signaling is an important modulator of p53 activation.

### Mtb-increased p53 activation is related to the anti-mycobacterial effects of M1 macrophages

**Fig. 3:**
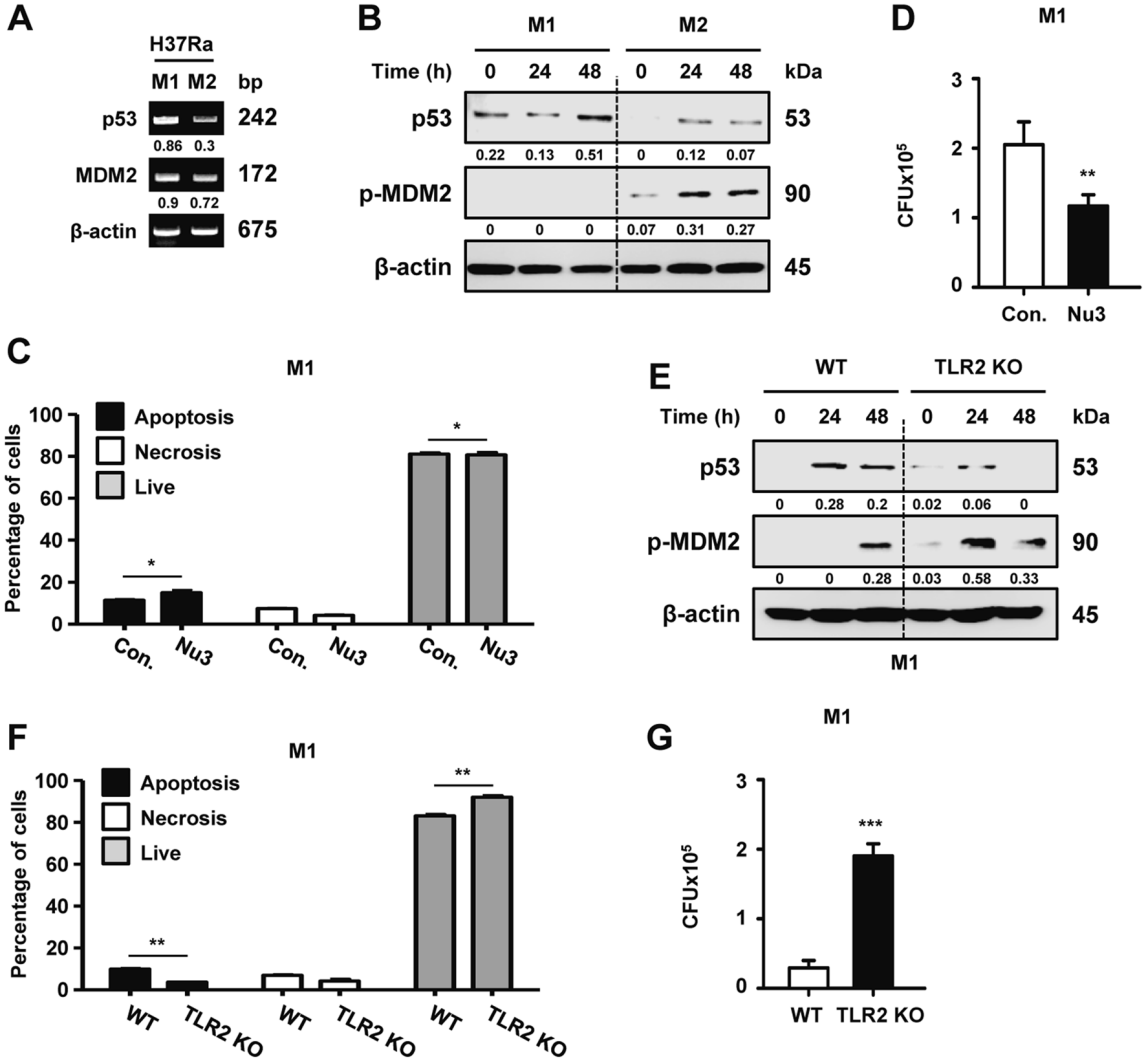
Activation of p53 is related to the anti-mycobacterial effects of M1 macrophages via the TLR2 signaling pathway. BMDMs were polarized to the M1 and M2 phenotypes and then **a** the mRNA expression and **b** protein levels of p53 and MDM2 were analyzed. **c** Nutlin-3-treated M1 macrophages were measured for H37Ra infection-induced apoptotic cell death using Annexin V-PI staining. **d** M1 macrophages were infected with H37Ra, treated with nutlin-3 (10 μM), and measured for intracellular survival after infection for 48 h. **e** M1 macrophages from WT and TLR2 KO mice were infected with H37Ra and were analyzed for the activation of p53 and MDM2 using Western blotting. **f** TLR2 KO-M1 macrophages were measured for H37Ra infection-induced apoptotic cell death using Annexin V-PI staining. **g** Intracellular Mtb survival was determined in WT-M1 and TLR2 KO-M1 macrophages after H37Ra infection for 48 h. All data are representative of three independent experiments. Statistically significant differences are indicated as follows: *p < 0.05, **p < 0.01, and ***p < 0.001

In a previous study, we showed that the TLR2 signaling pathway promotes the inflammatory response and ER stress-mediated apoptosis in M1 macrophage polarization by Mtb infection [6]. Mtb infection changes the macrophage phenotype from the M1 to the M2 type, which depends on virulent factors of Mtb such as ESAT-6 [6]. Because p53 expression is increased more in H37Ra-infected macrophages than in H37Rv infection, we hypothesized that attenuated H37Ra infection polarizes macrophages toward the M1 type, a phenomenon that is associated with p53 activation and p53-dependent anti-mycobacterial effects in infected macrophages. To address the relationship between macrophage polarization and p53 regulation, we first checked H37Ra-mediated p53 activation in M1- or M2-polarized macrophages. The expression of p53 mRNA and protein was significantly induced in Mtb-infected M1 macrophages compared to that in M2 macrophages (Fig. 3a, b). MDM2 expression, on the other hand, was higher in M2 macrophages than in M1 macrophages during Mtb infection. To confirm that changes in the macrophage subtype are p53-dependent, we checked the expression of inducible nitric oxide synthase (iNOS; marker for M1 macrophage) and arginase 1 (marker for M2 macrophage) in M1/M2-polarized macrophages from p53 WT and LysM-Cre;p53^flox/flox^ mice. As shown in Figure S3a, p53 deficiency was not associated with macrophage polarization during Mtb infection. Likewise, nutlin-3-induced p53 activation in the M1 types did not affect the modulation of macrophage polarization during Mtb infection (Fig. S3b). Induction of p53 and macrophage polarization were not changed by treatment with nutlin-3 in M2 macrophages (Fig. S3c). In addition, p53 activation elicited by nutlin-3 treatment in M1 macrophages enhanced the apoptotic cell ratio (Fig. 3c) but did not affect the induction levels of apoptosis in M2 macrophages (Fig. S3d, e). Importantly, nutlin-3 treatment resulted in the suppression of the intracellular survival of H37Ra in both M1 and M2 macrophages (Figs. 3d, S3f).

Next, we checked TLR2-dependent p53 activation in M1-polarized macrophages during H37Ra infection. Notwithstanding skewing toward M1 macrophages, the absence of TLR2 resulted in significantly lower levels of p53 protein in M1 macrophages (Fig. 3e). Furthermore, apoptosis was significantly lower in TLR2-deficient M1 macrophages than in WT controls (Fig. 3f). Subsequently, increased intracellular survival of Mtb was observed at 48 h in TLR2 KO BMDMs (Fig. 3g). Regardless of the M1 or M2 macrophage subtype, the survival of Mtb was reduced in TLR2 KO BMDMs (Fig. S3g). Collectively, these data suggest that TLR2-dependent p53 activation is essential for the clearance of intracellular Mtb in M1 macrophages.

### p53 activation in M1 macrophages is closely associated with JNK activation

**Fig. 4:**
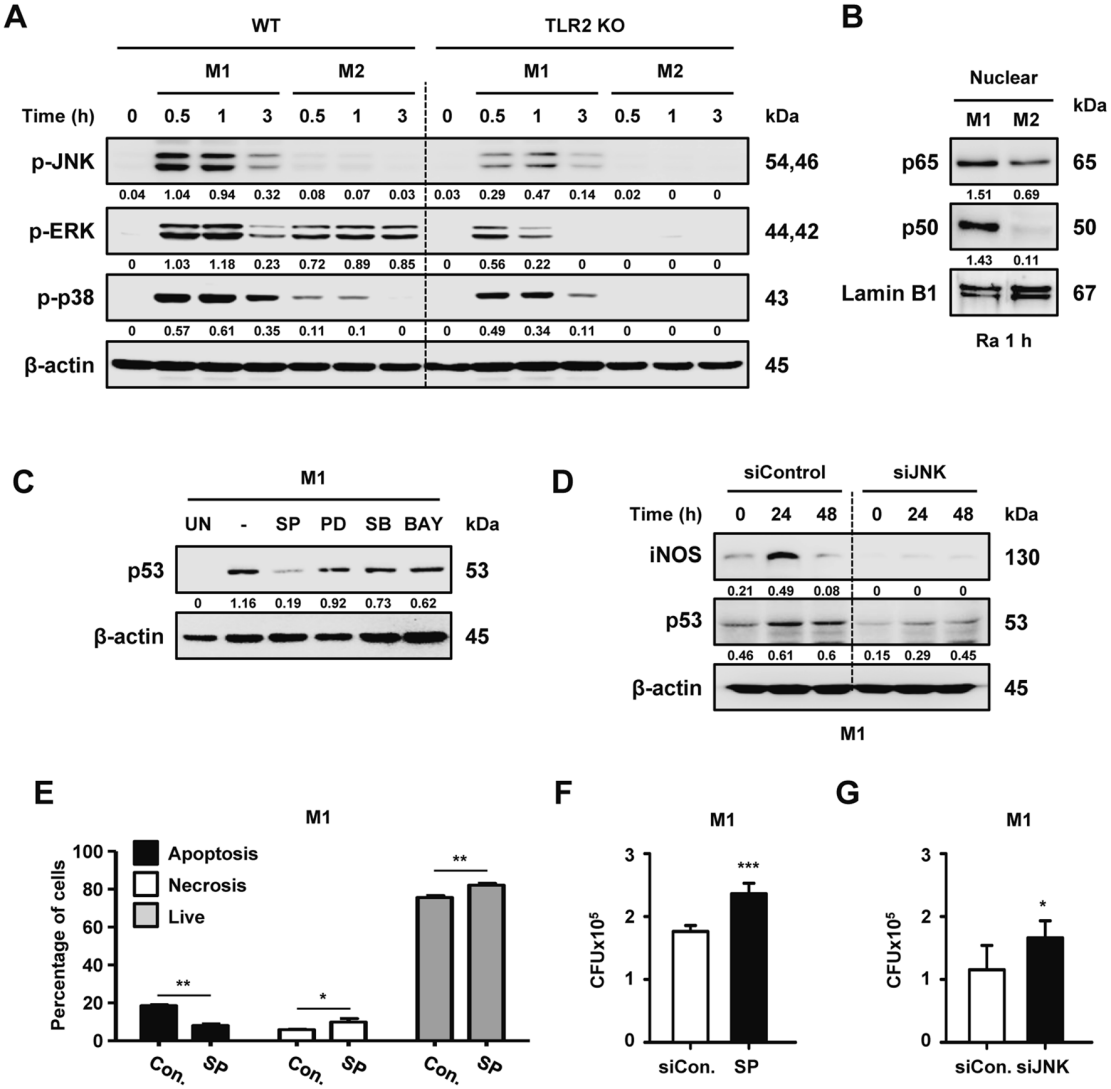
TLR2-dependent p53 expression in M1 macrophages is associated with JNK activation. WT and TLR2 KO BMDMs were polarized to M1 or M2 types and were infected with H37Ra for the indicated times. Activation of **a** JNK, ERK, and p38, as well as **b** NF-κB was determined using Western blotting. **c** M1-polarized macrophages pretreated with a specific inhibitor of JNK (SP600125, 30 μM), ERK (PD098059, 50 μM), p38 (SB203580, 10 μM), and NF-κB (BAY11-7082, 1 μM) for 2 h were infected with H37Ra for 48 h and then p53 protein expression levels were analyzed. **d** BMDMs were transfected with negative control or JNK siRNA (200 nM) for 18 h and then were polarized toward M1. After incubation for 24 h, these cells were infected with H37Ra. Expression of iNOS and p53 were analyzed by Western blotting. **e** H37Ra-induced apoptosis was measured in M1 macrophages pretreated with or without the JNK inhibitor. **f** JNK inhibitor-treated or **g** JNK siRNA-transfected M1 macrophages were assayed for intracellular Mtb survival using CFU analyses. All of the data are representative of three independent experiments. Statistically significant differences are indicated as follows: *p < 0.05, **p < 0.01, and ***p < 0.001

To explore whether TLR2-mediated MAPK activation is involved in M2 macrophage polarization, we first examined the activation of JNK, ERK, and p38 in H37Ra-infected M1 macrophages from WT and TLR2-deficient mice. As expected, the phosphorylation of each was higher in M1 polarized macrophages than in M2 macrophages (Fig. 4a). H37Ra-induced MAPK activation was not significantly different between WT and p53-deleted M1 macrophages (Fig. S4a). Next, we investigated NF-κB pathway because NF-κB pathway activation via TLR2 pathway is important to induce cytokine production during Mtb infection [32]. The nuclear translocation of p50 and p65 subunits (well-known markers for NF-κB activation) was also significantly higher in M1 macrophages than in M2 types during H37Ra infection (Fig. 4b). Only the JNK inhibitor remarkably reduced the production level of p53 in H37Ra-infected M1 macrophages (Fig. 4c). When macrophages were transfected with JNK small interfering RNA (siRNA) during H37Ra infection (Fig. S4b, c), the activation of iNOS and p53 was dramatically decreased in M1 macrophages compared to control macrophages (Fig. 4d). Similarly, JNK-mediated p53 activation was induced in H37Ra-infected WT MEF cells but not in H37Ra-infected JNK KO cells (Fig. S4d). These observations suggest that JNK signaling plays an important role in the regulation of macrophage polarization toward M1 via p53 activation. Decreased JNK activation led to dramatically lower levels of apoptotic cell death (Fig. 4e), and triggered enhanced levels of intracellular survival of H37Ra in M1 macrophages (Fig. 4f, g) but not in M2 macrophages (Fig. S4e). These data suggest that JNK-dependent M1 polarization plays an important role in the removal of mycobacteria via p53-induced apoptosis by macrophages.

### p53 activation in M1 occurs though the production of NO, ROS and inflammatory cytokines

**Fig. 5:**
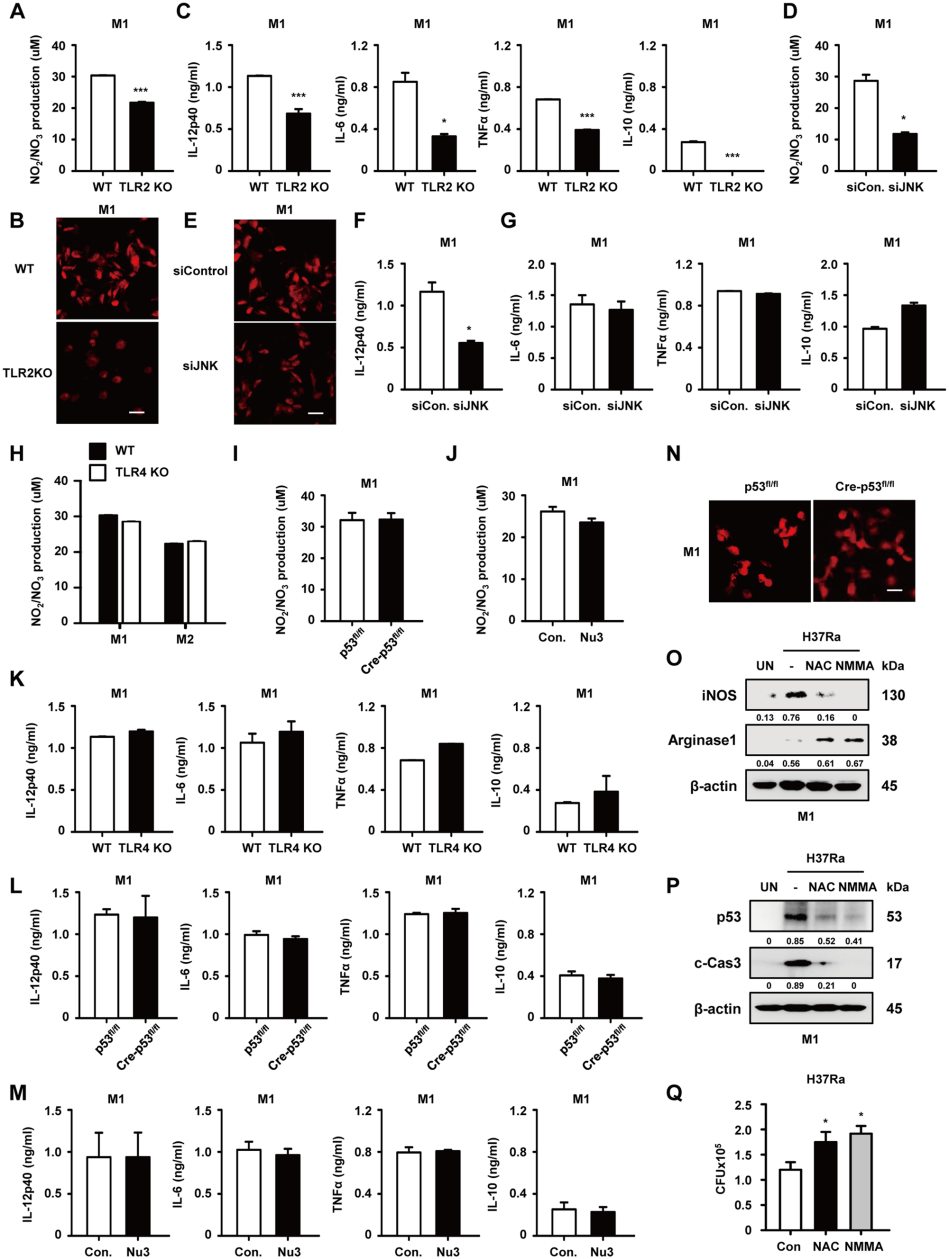
Activation of p53 in M1 occurs though TLR2-mediated production of NO, ROS and inflammatory cytokines. NO generation was measured using the Griess assay with culture supernatants from **a** WT- and TLR2 KO-M1 macrophages after H37Ra infection for 24 h. **b** ROS production caused by H37Ra infection for 30 min was detected in WT- and TLR2 KO-M1 macrophages via DHE (red) staining. **c** Cytokine secretion was evaluated by ELISA in H37Ra-infected M1 macrophages from WT and TLR2 KO mice. **d** Control siRNA- and JNK siRNA-transfected M1 macrophages were infected with H37Ra for 24 h, and NO production was measured. **e** ROS generation was detected in Control siRNA- and JNK siRNA-transfected M1 macrophages during H37Ra infection for 30 min. **f** Control siRNA- and JNK siRNA-transfected M1 macrophages were infected with H37Ra for 48 h, and secreted IL-12 in these cell supernatants was evaluated by ELISA. **g** Cytokine secretion in culture supernatants from control siRNA and JNK siRNA-transfected M1 macrophages were measured using ELISA. NO production was measured in **h** M1/M2 macrophages from WT and TLR4 KO mice, **i** p53-deleted M1 macrophages, or **j** nutlin-3-treated M1 macrophages during H37Ra infection for 24 h. **k** WT and TLR4 KO-M1 macrophages, **l** p53-deleted M1 macrophages, or **m** nutlin-3-treated M1 macrophages were subjected to H37Ra infection for 48 h, and cytokine secretion was measured. **n** ROS production after H37Ra infection for 30 min was detected using DHE (red) staining in WT and p53-deficient macrophages. M1 macrophages were pretreated with NAC (20 μM) or L-NMMA (1 μM) for 2 h and infected with H37Ra for 48 h. These cell lysates were analyzed for levels of **o** iNOS, arginase 1, **p** p53, and caspase-3 using Western blotting. **q** NAC or L-NMMA-treated M1 macrophages were assayed for intracellular Mtb survival using CFU analyses. Scale bar, 10 μm. All data are representative of three independent experiments. Statistically significant differences are indicated as follows: *p < 0.05, **p < 0.01, and ***p < 0.001 (Color figure online)

The induction of NO, ROS, and pro-inflammatory cytokines produced by activated immune cells leads to DNA damage, oxidative stress, and continuous inflammatory stress, as well as p53 activation [33]. Thus, we confirmed the production of each in H37Ra-infected M1 macrophages from WT and TLR2 KO mice. The absence of TLR2 signaling in M1 macrophages was associated with lower levels of NO (Fig. 5a) and ROS (Fig. 5b) during Mtb infection. In addition, the production of inflammatory cytokines such as IL-12, TNFα, IL-6, and IL-10 was reduced in TLR2 KO macrophages compared to WT controls (Fig. 5c). To further investigate the role of JNK signaling in NO and ROS production, we silenced JNK expression using JNK-specific siRNA. This led to reductions in the generation of NO (Fig. 5d) and ROS (Fig. 5e). Interestingly, IL-12 production was lower in Mtb-infected M1 macrophages pretreated with siJNK (Fig. 5f). However, the production of TNFα, IL-6, and IL-10 was not affected by siJNK (Fig. 5g). TLR4-deficient, myeloid p53-deficient, and nutlin-3-treated M1 macrophages did not alter NO production (Fig. 5h–j) or secreted cytokines (Fig. 5k–m) by H37Ra infection. In addition, ROS generation in H37Ra-infected M1 macrophages did not depend on p53 (Fig. 5n). To confirm the role of ROS and NO in the polarization of macrophages and activation of p53, we evaluated the expression of M1 and M2 markers and activation of p53 in the presence of NAC (ROS scavenger) and L-NMMA (NOS inhibitor). As expected, iNOS expression was lower in M1 macrophages pretreated with ROS or NO scavengers following H37Ra infection (Fig. 5o). In M2 macrophages, arinase1 was induced during ROS or NO scavengers treatment (Fig. S4f). By contrast, arginase 1 expression was induced in such macrophages, as were H37Ra-induced p53 and caspase-3 activation (Fig. 5p). In addition, intracellular survival of H37Ra was increased in presence of NAC and L-NMMA comparing to the control (Fig. 5q). These results indicate that the production of NO and ROS during Mtb infection leads to p53 expression through TLR2-JNK signaling activation in M1 macrophages.

### p53 activation controls intracellular Mtb survival in the lungs of mice and in the monocyte-derived macrophages (MDMs) of TB patients

**Fig. 6:**
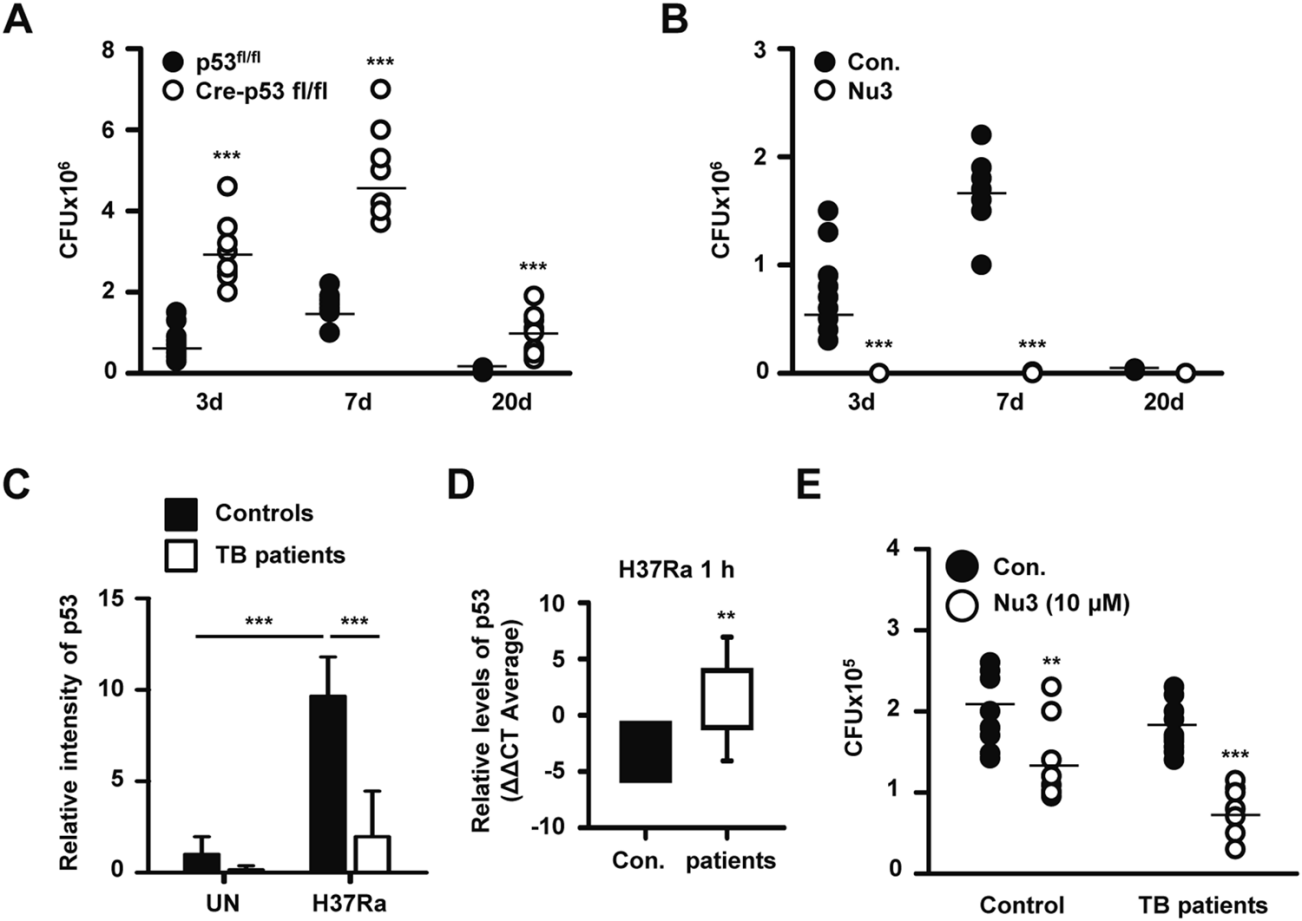
p53-mediated apoptosis effectively eliminates intracellular Mtb in vivo and in the MDMs of TB patients ex vivo. **a** WT (p53^flox/flox^) and Cre-p53^flox/flox^ mice were infected intranasally with H37Ra (1 × 10^6^) for 3, 7, and 20 days, and then the Mtb burden was determined in the lungs of infected mice by CFU enumeration. **b** H37Ra-infected mouse models, which were injected intraperitoneally with nutlin-3 (10 mg/kg/day) or PBS, were measured for the bacterial burden in lungs. The data reflect five mice per time point for each group, and are representative of three independent experiments. Blood MDMs isolated from healthy controls and TB patients were infected with H37Ra for 1 h, and the relative expression of p53 mRNA was measured. **c** The bar graph presents the quantitative data. Densitometric analyses of the detected bands by reverse transcription–PCR are shown in the bar graph. **d** Delta-delta CT values of p53 mRNA expression in MDMs of controls vs. TB patients were analyzed using quantitative real-time PCR. **e** H37Ra-infected MDMs from controls and TB patients were treated with nutlin-3 (10 μM). After incubation for 48 h, intracellular Mtb survival was measured in infected cells. All data are representative of three independent experiments. Statistically significant differences are indicated, as follows: *p < 0.05, **p < 0.01, and ***p < 0.001

To further extend our in vitro findings, we confirmed the antibacterial role of p53 during Mtb infection in vivo. WT and myeloid p53-deleted mice were infected with H37Ra intranasally and then were analyzed for bacterial burdens in the lung after 3, 7, and 20 days. Bacterial loads were higher in the lungs of infected mice 3 days after infection and even higher after 7 days. In addition, Mtb survival gradually decreased over 20 days both in both groups. In agreement with our in vitro observations, the p53 KO mouse group showed significantly increased intracellular survival of H37Ra (n = 10, 4.84 × 10^6^ CFU/lung) compared to WT controls (n = 10, 1.62 × 10^6^ CFU/lung) at day 7 (Fig. 6a). Indeed, H37Ra was almost eliminated in lung tissues by nutlin-3 treatment 3 days after infection (Fig. 6b). These data indicate that p53 activation plays an important role in reducing Mtb survival. To confirm the function of p53 in macrophages from TB patients, we first examined H37Ra infection-induced p53 expression in human MDMs obtained from healthy individuals and TB patients. Interestingly, p53 mRNA was highly expressed in healthy controls but not in TB patients (Figs. 6c; S6a, b). Real-time polymerase chain reaction (PCR) analyses indicated that p53 mRNA expression levels were lower in TB patients (the delta-delta CT values of p53 mRNA were − 3.262 ± 1.396 in healthy controls and 1.441 ± 5.499 in TB patients; p = 0.0033) (Fig. 6d). These results suggest that decreased p53 activation in TB patients promotes Mtb survival. Corroborating these findings, we observed that nutlin-3 effectively abrogated the intracellular survival of mycobacteria in both TB patients and healthy controls after H37Ra infection for 24 h (Fig. 6e). Nutlin-3 treatment induced p53 mRNA expression in H37Ra-infected MDMs (Fig. S6c). Overall, these findings suggest that p53-induced apoptosis plays an important role in the suppression of Mtb in hosts.

## Discussion

**Fig. 7:**
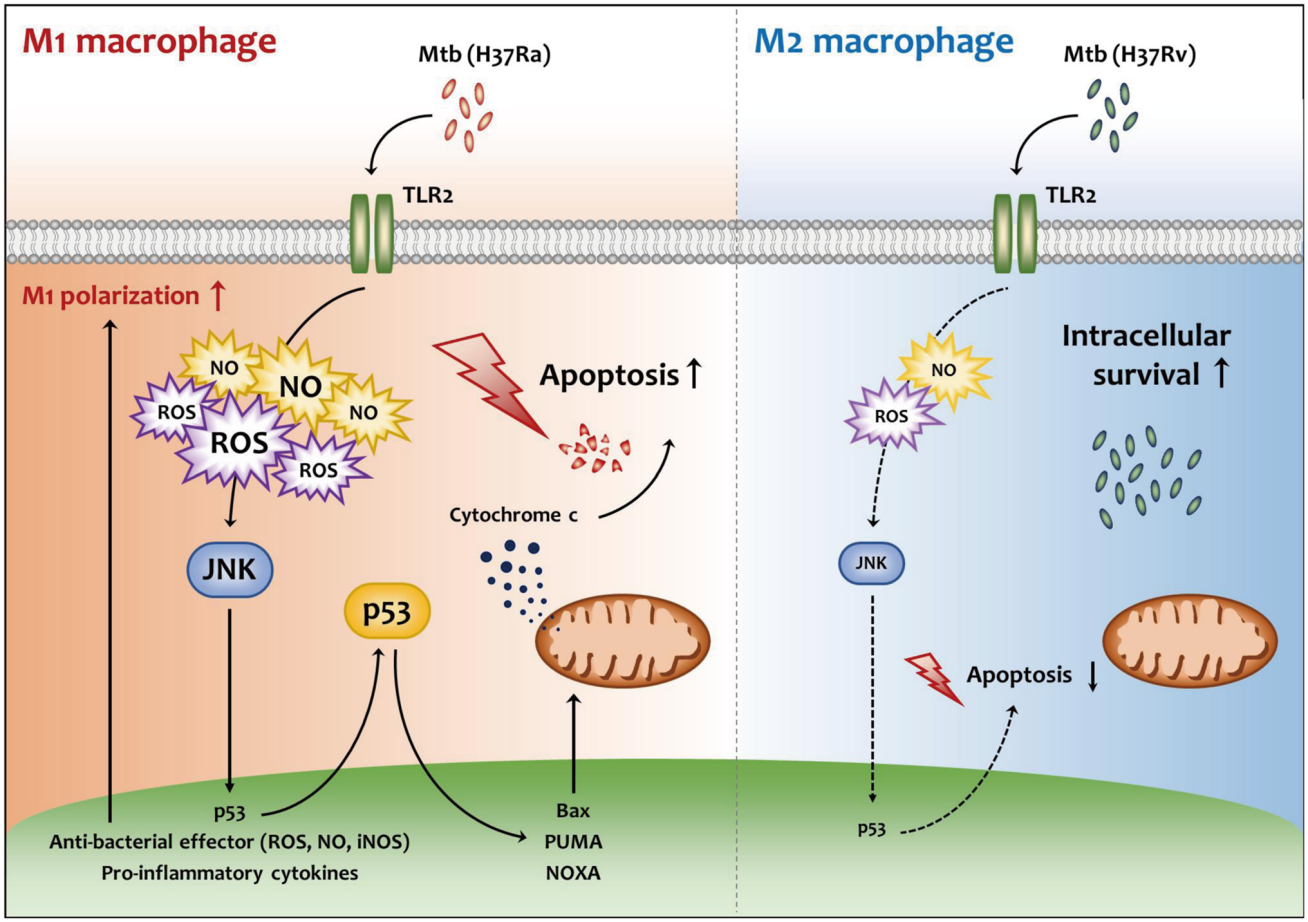
Schematic diagram depicting the mechanism of TLR2/JNK/p53-mediated apoptosis in M1 macrophages during Mtb infection. In M1 macrophages, the induction of ROS and NO through TLR2 activates JNK/p53 pathway during Mtb infection. The increased p53 induces pro-apoptotic signal including PUMA, NOXA and Bax leading to apoptosis. Whereas, in M2 macrophages, TLR2/JNK/p53 pathway is decreased comparing to M1 macrophages during Mtb infection. TLR2/JNK/p53-mediated apoptosis in M1 macrophages plays an important role in removing intracellular Mtb

The p53 signaling pathway plays an important role in the induction of apoptosis. Likewise, the apoptosis of Mtb-infected macrophages is required to diminish mycobacterial viability [10]. A previous report showed that p53 is closely associated with increased levels of TNFα during Mtb infection [30]. Therefore, we predicted that p53-induced apoptosis may have important functions in Mtb-infected cells. In our study, we clearly showed that caspase activation-dependent apoptosis release and its bactericidal effect against H37Ra are reduced in the absence of p53 in macrophages. However, enhanced activation of p53 by nutlin-3 treatment led to increased macrophage apoptosis and bacterial clearance. Specifically, we found that treatment of nutlin-3 significantly reduced intracellular H37Ra in mice lung tissues, MDMs of TB patients and healthy controls (Fig. 6b, e). Our results provide the first evidence that p53 effectively prevents attenuated H37Ra survival in M1-polarized macrophages by activation of the apoptotic pathway (Fig. 7).

The activity of p53 is modulated by interactions with the E3 ligase MDM2, which binds stably to p53, leading to its degradation [34]. We found that MDM2 protein expression was upregulated in H37Rv-infected macrophages compared to H37Ra-infected cells, and was upregulated to a greater extent in Mtb-infected M2 macrophages than in M1 types. Indeed, p53 signaling can regulate M2 macrophage polarization. Earlier studies have shown that p53 activated by nutlin-3 treatment powerfully decreases the expression of M2-associated genes [35], resulting in the suppression of M2-like tumor-associated macrophages (TAMs) [36, 37]. Meanwhile, the functions of p53 are disrupted in TAMs, which promotes invasion, metastasis, proliferation, and survival [38]. Similarly, virulent H37Rv infection can skew toward M2 macrophages and in turn suppress p53 by inducing MDM2 activation. Of note, nutlin-3 treatment also effectively decreased intracellular Mtb in M2-polarized macrophages, although the M2 phenotypes benefit bacterial survival. These results suggest that M2-skewed polarization suppresses p53-mediated cell death via MDM2 induction, which might be considered a unique survival strategy of Mtb. Therefore, p53 activation could be a good strategy for TB treatment.

The balance between M1 and M2 macrophage polarization plays an important role in TB [39]. The macrophage polarization ratio determines immune functions, including inflammation and antimicrobial activity within the granuloma model, and NF-κB signaling is particularly important to promote M1 polarization during early TB infection [40]. Most discussions concerning the relationship between macrophage polarization and p53 have focused on their roles in cancer [35, 36, 41, 42], but not in infectious diseases such as TB. Previously, we suggested that ER stress-mediated apoptosis in M1 macrophages is important and required for the elimination of intracellular Mtb [6]. M1 macrophages highly promote the generation of ROS/NO and secretion of pro-inflammatory cytokines, which are well known activators of the ER stress response. In addition to the ER stress response, p53-dependent signaling is regulated by ROS/NO production or inflammation. Here, we note that p53 activation in M1-polarized macrophages plays critical roles in the apoptosis-mediated bactericidal effect against Mtb, an effect that is similar to the ER stress response. It is important to determine the effective defense mechanism against Mtb in M1 phenotypes.

p53 modulates inflammation and immune responses resulting from the production of inflammatory cytokines via activation of the NF-κB and MAPK pathways [43, 44]. In addition, it is activated by the increased generation of ROS and reactive nitrogen species (RNS) during inflammation [45, 46]. We propose that p53 activation in Mtb-infected macrophages is involved in the TLR2 signaling pathway, which triggers both NF-κB and MAPK activation. We suggest that activation of the NF-κB and MAPK pathways during Mtb infection contributes to the production of ROS/NO and inflammatory cytokines including IL-12, IL-6, and TNFα, and that such stressful stimuli might lead to p53-mediated apoptosis via MAPK activation. Furthermore, recent studies have shown that NO- or ROS-related mechanisms in macrophages are used for host defense and inflammatory responses in various diseases. In particular, M1 macrophages produce NO, ROS, and pro-inflammatory cytokines at high levels compared to M2 types [47]. Thus, increased production of NO, ROS, and pro-inflammatory cytokines in M1 macrophages likely has a positive influence on p53 activation during Mtb infection.

We observed that p53 activation in M1 polarization is tightly linked to the JNK-dependent pathway. The expression levels of iNOS and M1-related p53 activation were decreased by the JNK specific inhibitor or JNK siRNA transfection even when macrophages were treated with M1-polarizing stimuli. Our findings are consistent with the literature; most recent studies have reported that JNK activation is an essential factor driving M1 macrophage polarization via the secretion of pro-inflammatory cytokines [48–51]. Moreover, signal transducer and activator of transcription 1 (STAT1) has emerged as a major transcriptional factor in M1 polarization. By contrast, ERK1/2, STAT3, and STAT6 signaling are associated with TAMs [52]. Furthermore, Mtb-infected macrophages promote anti-inflammatory responses via TLR2-dependent ERK signaling activation [53]. It is well known that the MEKK1/JNK signaling is important for stabilization and activation of p53 to mediate apoptosis [54]. Similarly, our results showed that p53-mediated apoptosis depends on JNK signaling activation in M1 types. Importance of dynamic epigenetic regulation during monocyte to macrophage differentiation has been suggested after infection or vaccination [55]. Macrophage activation can be trained by environmental signals such as cytokines and microbial components [55]. Based on our observations, p53 signaling pathway is important for the protective effects of trained immunity during mycobacterial infection. Although p53 was not directly associated with macrophage polarization during Mtb infection, but we found that p53 expression was increased in Mtb-infected M1 macrophages though TLR2-JNK pathway. Thus, it is estimated that the apoptotic machinery of JNK-dependent p53 activation in M1 might be a promising strategy for the inhibition of Mtb survival.

## Materials and Methods

### Mice and cells

Pathogen-free female WT, TLR4-deficient, TLR2-deficient, MyD88-deficient, p53^flox/flox^, and LysM-Cre;p53^flox/flox^ conditional knockout mice (C57BL/6 background) were maintained in specific-pathogen-free conditions and used at 6–8 weeks of age in all experiments. BMDMs were isolated and polarized to M1 or M2 types as described previously [6]. Briefly, BMDMs were isolated and differentiated for 4–5 days in Dulbecco’s minimal essential medium (DMEM) containing 10% fetal bovine serum (FBS), penicillin (100 IU/mL), streptomycin (100 μg/mL), and 25 ng/mL macrophage colony-stimulating factor (M-CSF; R&D Systems, Minneapolis, MN, USA). For M1 polarization, macrophages were incubated with 10 ng/mL lipopolysaccharide (LPS; InvivoGen, San Diego, CA, USA) plus 10 ng/mL mouse IFN-γ (R&D Systems) for 24 h. For M2, cells were incubated with 10 ng/mL mouse IL-4 (R&D Systems) plus 10 ng/mL mouse IL-13 (R&D Systems) for 24 h. WT and JNK-/- mouse embryonic fibroblasts (MEFs) were cultured in DMEM containing 10% FBS, penicillin and streptomycin at 37 °C and 5% CO2. Human PBMCs were isolated from heparinized venous blood using Lymphoprep (Axis-Shield, Dundee, UK) as described previously [56]. For macrophage differentiation, adherent monocytes were incubated in RPMI 1640 with 10% pooled human serum, 1% l-glutamine, 50 IU/ml penicillin, and 50 μg/ml streptomycin for 1 h at 37 °C, and nonadherent cells were removed. Human MDMs were prepared by culturing peripheral blood monocytes for 4 days in the presence of 4 ng/ml human CSF/macrophage colony-stimulating factor (Sigma-Aldrich, St. Louis, MO, USA) as described previously [57].

### Patients

Blood samples were collected from 9 healthy controls and 13 patients with active TB. The mean age of TB patients (male: n = 7; female: n = 6) was 56.153 ± 22.345 years, and that of the healthy group (male: n = 8, female: n = 1) was 28.111 ± 7.975 years. All patients were newly diagnosed with TB disease during 2017–2018, and blood samples were collected from patients before treatment began.

### Mtb infection and intracellular survival analyses in vitro and in vivo

Mycobacterial culture and in vitro macrophage infection were performed as described previously [14]. Briefly, the Mtb strain H37Ra (ATCC 25177) was grown in Middlebrook 7H9 liquid medium supplemented with 10% OADC (oleic acid, albumin, dextrose, catalase) and 5% glycerol and then was suspended in phosphate-buffered saline (PBS) at a concentration of 1 × 10^8^ CFU/mL. Cells were infected with live at an MOI of 1, and were incubated for 3 h at 37 °C, 5% CO2. After allowing time for phagocytosis, cells were washed with PBS to remove extracellular bacteria and then were incubated with fresh medium without antibiotics for an additional 24 or 48 h. In vivo mice were challenged by intranasal infection with Mtb (1 × 10^6^ in 50 µL PBS) into the lungs. Then, 3, 7, and 20 days after infection, five mice per group were sacrificed in duplicate. To test the intracellular survival of Mtb in vitro and in vivo, infected cells or lung tissues from infected mice were lysed in sterile distilled water to allow intracellular bacteria to be collected. The lysates were serially diluted in 7H9 broth, plated separately onto 7H10 agar plates, and incubated for 2–3 weeks. Colonies were counted in triplicate.

### Antibodies, reagents and transfections

Cells were pretreated with inhibitors or inducers for 2 h prior to Mtb infection. Specific inhibitors of JNK (SP600125), ERK (PD098059), p38 (SB203580), and NF-κB (BAY11-7082) were purchased from Calbiochem (San Diego, CA, USA). The ROS scavenger (N-acetyl-l-cysteine; NAC) and NOS inhibitor (nitric oxide synthase inhibitor; L-NMMA) were purchased from Sigma-Aldrich. Nutlin-3 (Santa Cruz Biotechnology, Santa Cruz, CA, USA) was used as a p53 activator by MDM2 inhibition. Western blotting was performed using antibodies against phospho-MDM2, caspase-9, caspase-3, phospho-JNK, phosphor-ERK, phosphor-p38, p65, p50 (Cell Signaling, Danvers, MA, USA) p53, iNOS, arginase 1, β-actin, and laminB1 (Santa Cruz). Goat anti-rabbit IgG (Santa Cruz) and goat anti-mouse IgG (1:2000; Calbiochem) were used as secondary antibodies. Transfection of siControl (200 nM; Santa Cruz) and siJNK (200 nM; Bioneer Corporation, Daejeon, South Korea) into macrophage cells was performed using Lipofectamine 3000 (Invitrogen) according to the manufacturer’s instructions.

### PCR, Western blotting and enzyme-linked immunosorbent assay

Mtb-infected macrophages were processed by PCR, Western blotting, and sandwich enzyme-linked immunosorbent assay (ELISA) as described previously [6]. Briefly, total RNA was isolated from Mtb-infected BMDMs, and mRNA was reverse transcribed into cDNA. Reverse transcription–PCR was performed using Prime Taq Premix (Genet Bio, Daejeon, Korea) to detect the mRNA levels of target genes. For quantitative real-time PCR, total RNA from the MDMs of healthy controls and TB patients was extracted, cDNA was synthesized, and then p53 gene expression was quantified by SYBR green (Qiagen, Hilden, Germany). The mean value of triplicate reactions was normalized against the mean value of β-actin.

For Western blotting, Mtb-infected cells were lysed, and the lysates were separated by sodium dodecyl sulfate–polyacrylamide gel electrophoresis, followed by transfer to a polyvinylidene difluoride (PVDF) membrane. The membrane was blocked with 5% nonfat milk for 1 h at room temperature. Primary antibodies were diluted 1:1000, and horseradish peroxidase (HRP)-conjugated secondary antibodies were diluted 1:2000. For the detection of target proteins, the membranes were developed using a chemiluminescent reagent (ECL; Millipore) and were subsequently quantified using the Alliance Mini 4 M (UVITEC, Cambridge, UK).

The secretion levels of cytokines in the cell culture supernatants were measured using sandwich ELISA with detection kits for mouse IL-12p40, TNF, IL-6, and IL-10 (BD Biosciences, Franklin Lakes, NJ, USA). The sample absorbances were measured using a microplate reader at 450 nm and were compared to a standard curve.

### Apoptosis assay

To confirm the ratio of apoptotic cells, BMDMs were stained using an Annexin-V/PI staining kit (BD Biosciences) as described in the manufacturer’s instructions and then were analyzed using a FACS Canto II flow cytometer (BD Immunocytometry Systems, Franklin Lakes, NJ, USA).

### ROS and NO measurement assays

To detect intracellular ROS production, Mtb-infected BMDMs were measured using the dihydroethidium (DHE) assay. Macrophages were infected with Mtb for 30 min, stained with 2 μM DHE for 30 min, and then washed with Krebs-Hepes buffer. Positive cells were identified using a laser-scanning confocal microscope (TCS SP8; Leica Microsystems, Wetzlar, Germany).

To evaluate NO levels during Mtb infection, macrophage cell culture supernatant fractions were analyzed using the Griess assay. Briefly, culture medium (100 µL) was incubated with the Griess reagent (100 µL) at room temperature for 10 min, and then the absorbance was measured at 541 nm. Sodium nitrite was used to create a standard concentration curve.

### Data analysis and statistics

All experimental results were statistically evaluated using Student’s *t* test or one-way analysis of variance followed by Bonferroni’s multiple comparison tests. Statistical significance between groups was determined using the appropriate nonparametric Mann–Whitney or Kruskal–Wallis test. Differences were deemed significant when the p-value was < 0.05, and a difference of p < 0.001 was deemed highly significant. All experiments were performed three to five times, and the data are presented as means ± SDs. In vivo assays were performed in triplicate, and a minimum of three mice was used per group. Statistical analyses were performed using GraphPad Prism software (version 5.01).

## Electronic supplementary material

Below is the link to the electronic supplementary material.

Supplementary material 1 (DOCX 1346 kb)
