# Correction to: M1 macrophage dependent-p53 regulates the intracellular survival of mycobacteria

**DOI:** 10.1007/s10495-019-01581-5

**Published:** 2019-11-27

**Authors:** Yun-Ji Lim, Junghwan Lee, Ji-Ae Choi, Soo-Na Cho, Sang-Hun Son, Sun-Jung Kwon, Ji-Woong Son, Chang-Hwa Song

**Affiliations:** 1 Department of Microbiology, College of Medicine, Chungnam National University, Daejeon, 35015 South Korea; 2 Department of Medical Science, Chungnam National University, Daejeon, South Korea; 3 Research Institute for Medical Sciences, College of Medicine, Chungnam National University, Daejeon, South Korea; 4 Department of Internal Medicine, Konyang University Hospital, Daejeon, South Korea

## Correction to: Apoptosis 10.1007/s10495-019-01578-0

The original version of this article unfortunately contains an error in the acknowledgement section. The text “Brain Korea 21 PLUS Project for Medical Science, Chungnam National University” was omitted by mistake.

The correct and complete acknowledgment is given below:

