# Supplementary material for: M1 macrophage dependent-p53 regulates the intracellular survival of mycobacteria
Source: Apoptosis. 2019 Nov 5;25(1):42–55. doi: 10.1007/s10495-019-01578-0 (PMC6965052; doi:10.1007/s10495-019-01578-0)
Supplement: Supplementary file 1 — Supplementary material 1 (DOCX 1346 kb) [file 10495_2019_1578_MOESM1_ESM.docx]

**Supporting Information**


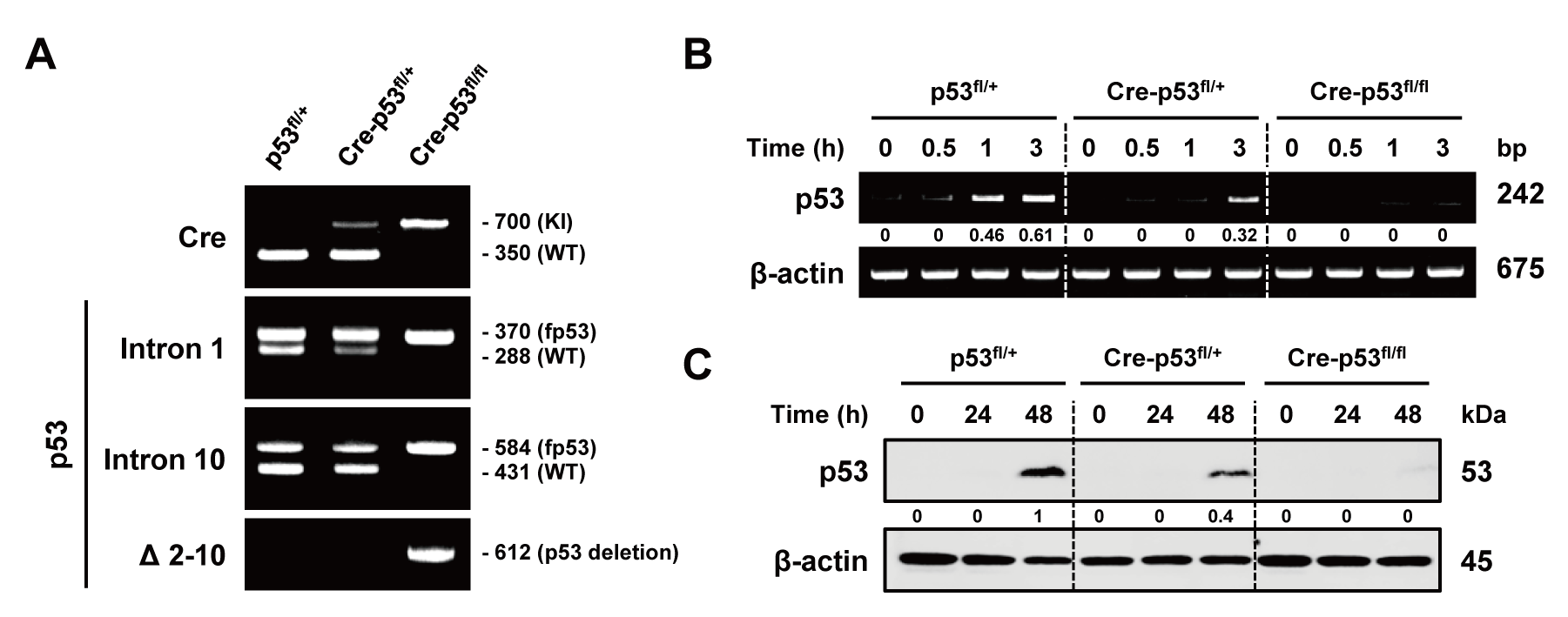


**Figure S1. Conditional inactivation of p53 in macrophages**

(A) WT (p53^flox/flox^ and Cre-p53^flox/+^) and Cre-p53^flox/flox^ mice genotypes were identified by PCR. The expression levels of (B) mRNA and (C) p53 protein were analyzed in BMDMs from these mice after H37 Ra infection at the indicated times.


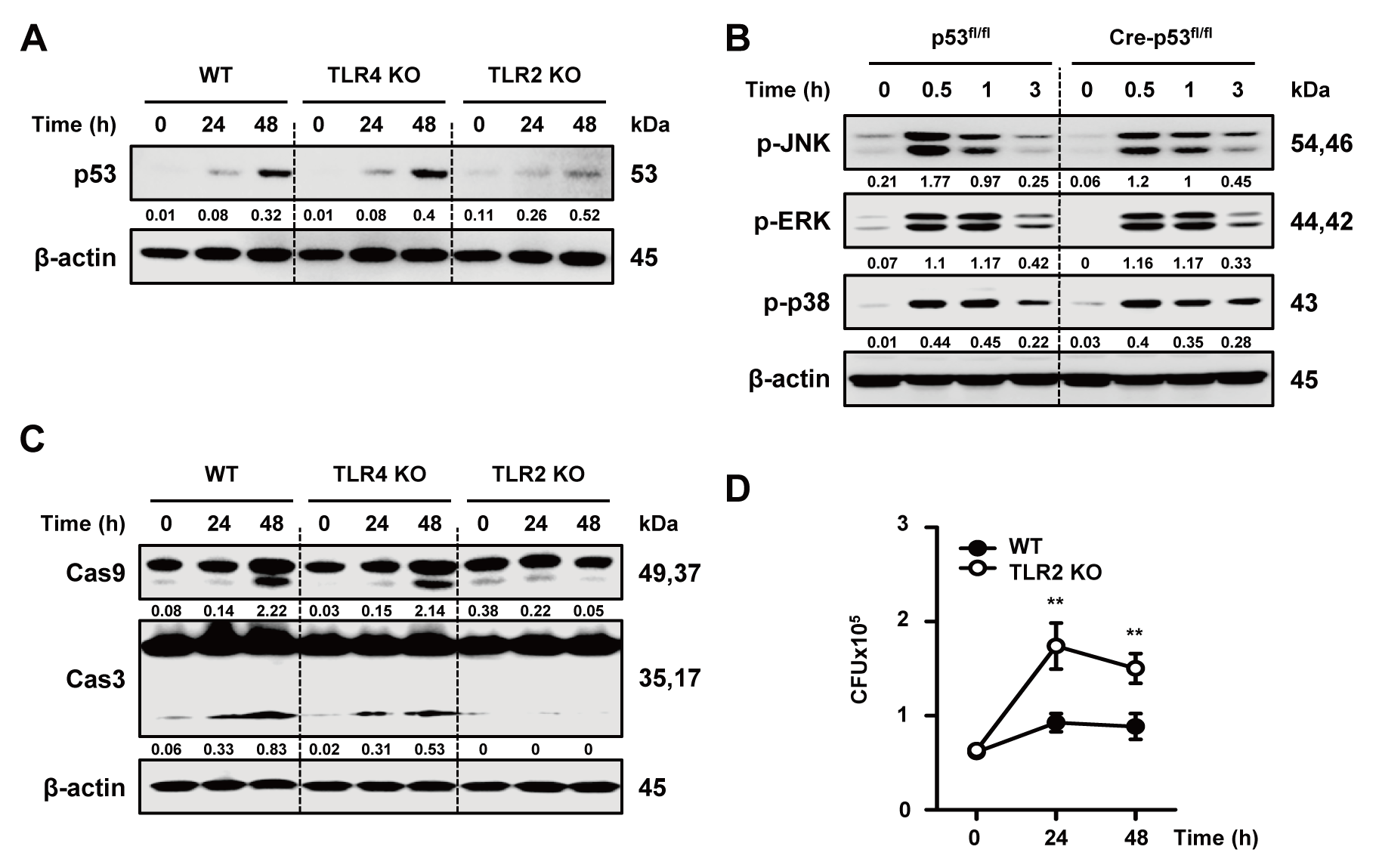


**Figure S2. TLR2-dependent signaling activation in Mtb-infected macrophages is associated with p53 activation**

WT and TLR4-, TLR2-, and MyD88-deficient BMDMs were infected with H37Ra and then analyzed for (A) p53 protein expression. (B) MAPK activation was measured in the BMDMs from WT and Cre-p53^flox/flox^ after H37Ra infection. (C) WT and TLR4-, TLR2-, and MyD88-deficient BMDMs infected with H37Ra were analyzed for caspase activation. (D) H37Ra-infected WT and TLR2-deficient cells were measured for the intracellular survival of Mtb by CFU enumeration. The results are representative of three independent experiments. Statistically significant differences are indicated; **p<0.01


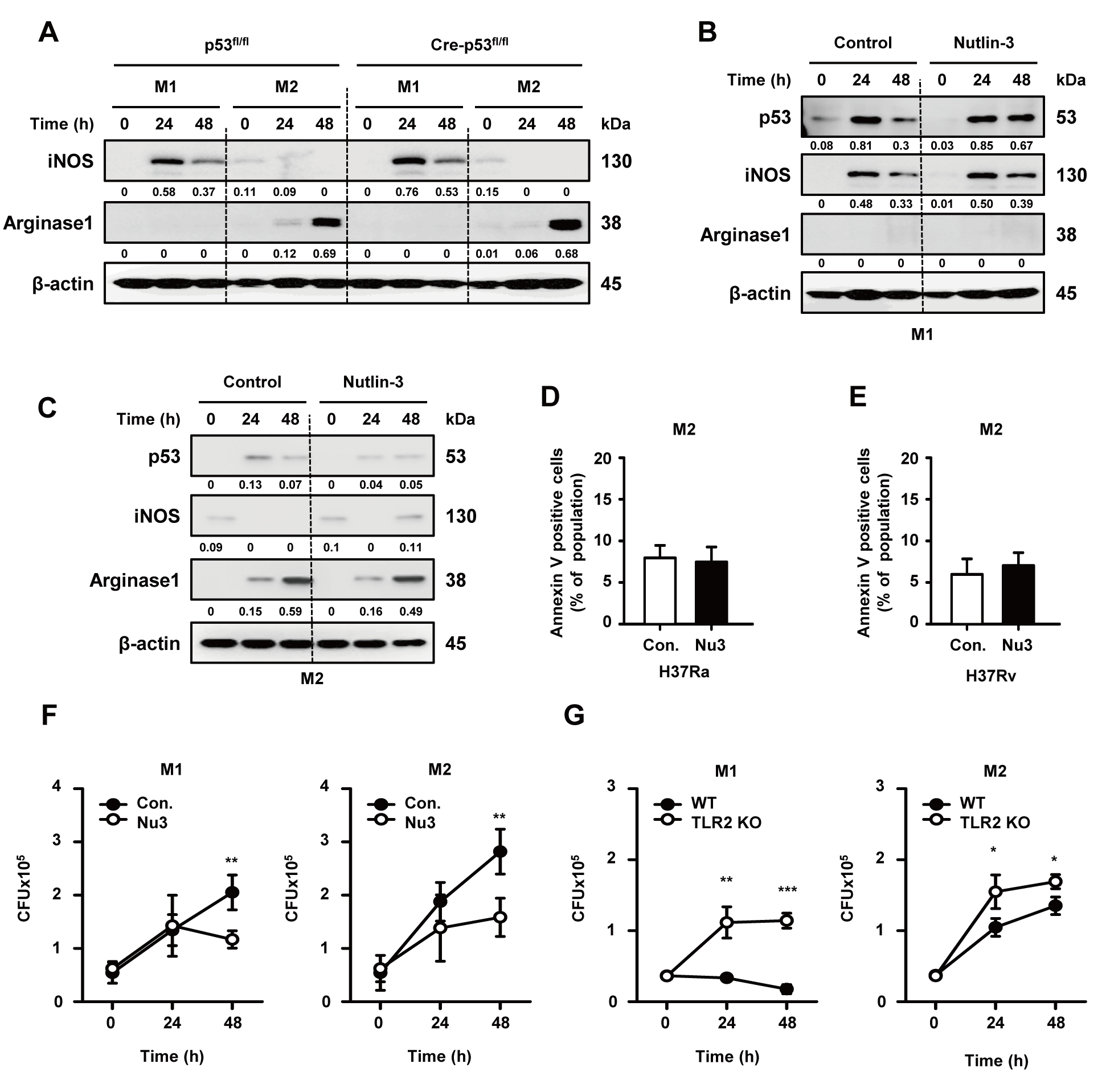


**Figure S3. Activation of p53 in M1 macrophages is essential for the anti-mycobacterial effect**

(A) The expression levels of iNOS and arginase 1 were measured in M1- or M2-polarized macrophages from WT and Cre-p53^flox/flox^ mice after H37Ra infection. In H37Ra-infected (B) M1 or (C) M2 macrophages, we measured the expression of p53, iNOS, and arginase 1 in the presence or absence of nutlin-3 (10 μM). In M2-polarzied BMDMs, we analyzed (D) H37Ra- or (E) H37Rv-induced apoptotic cell death via Annexin V-PI staining. (F) M1 or M2 macrophages from WT and TLR2 KO mice were infected with H37Ra and analyzed for intracellular Mtb survival after infection. (G) Intracellular Mtb survival was determined in M1 or M2 cells pretreated with nutlin-3. All data are representative of three independent experiments. Statistically significant differences are indicated, as follows: *p <0.05, **p <0.01, and ***p <0.001.


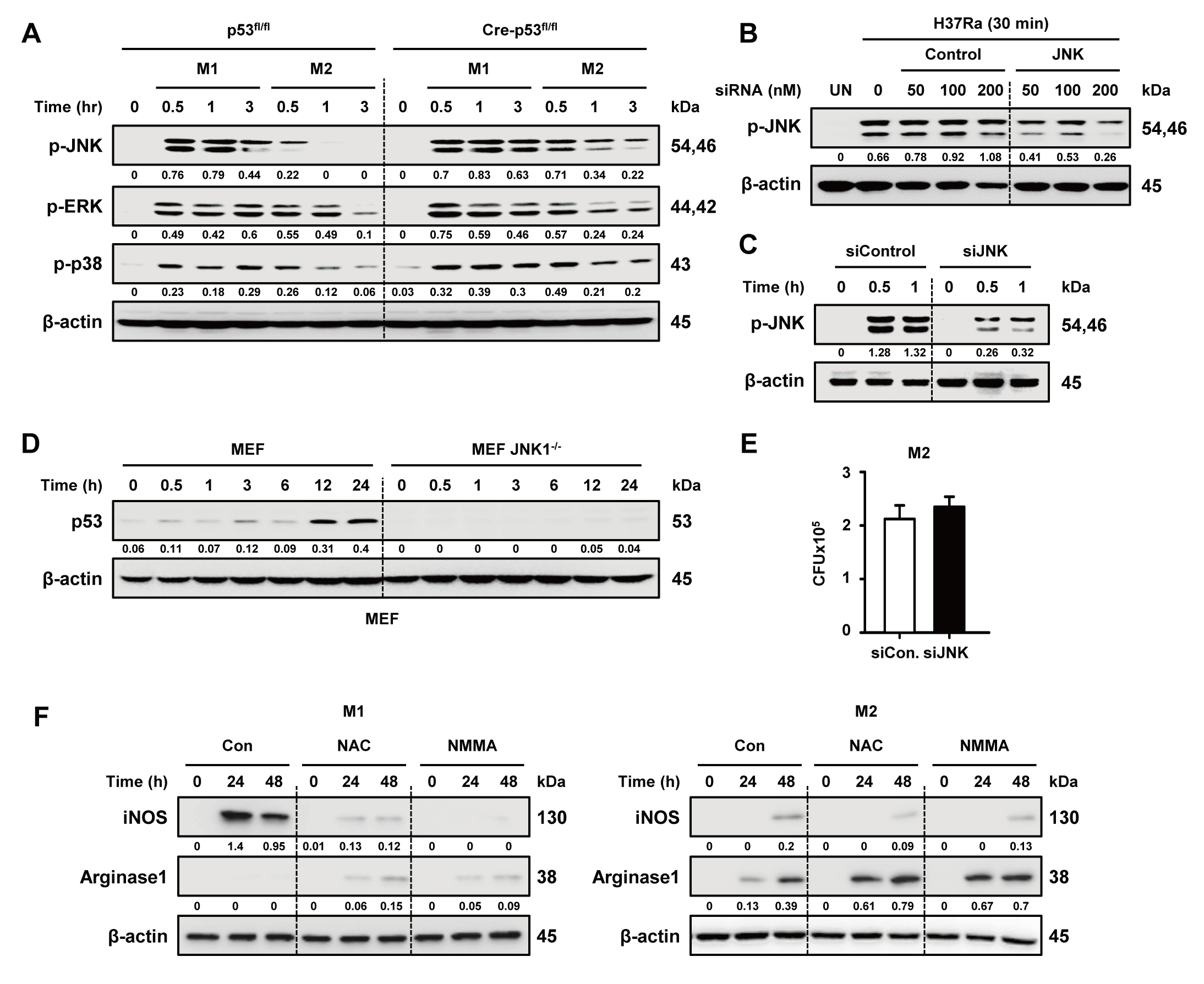


**Figure S4. M1-mediated JNK signaling is important to p53 expression**

(A) The activation of MAPK signaling was determined in WT and p53-deleted M1 or M2 macrophages during H37Ra infection. BMDMs transfected with Control siRNA or JNK siRNA were infected with H37Ra, and JNK phosphorylation was observed (B) following dose-dependent transfection or (C) time-dependent infection. (D) WT- and JNK1-deleted MEF cells were infected with H37Ra and then analyzed for p53 protein expression levels. (E) Control or JNK siRNA-transfected M2 macrophages were determined for intracellular Mtb survival by CFU analyses. (F) M1 or M2 macrophages were pretreated with NAC (20 μM) or L-NMMA (1 μM) for 2 h and infected with H37Ra for 48 h. These cell lysates were analyzed for levels of iNOS and arginase 1 using Western blotting. All data are representative of three independent experiments. Statistically significant differences are indicated.


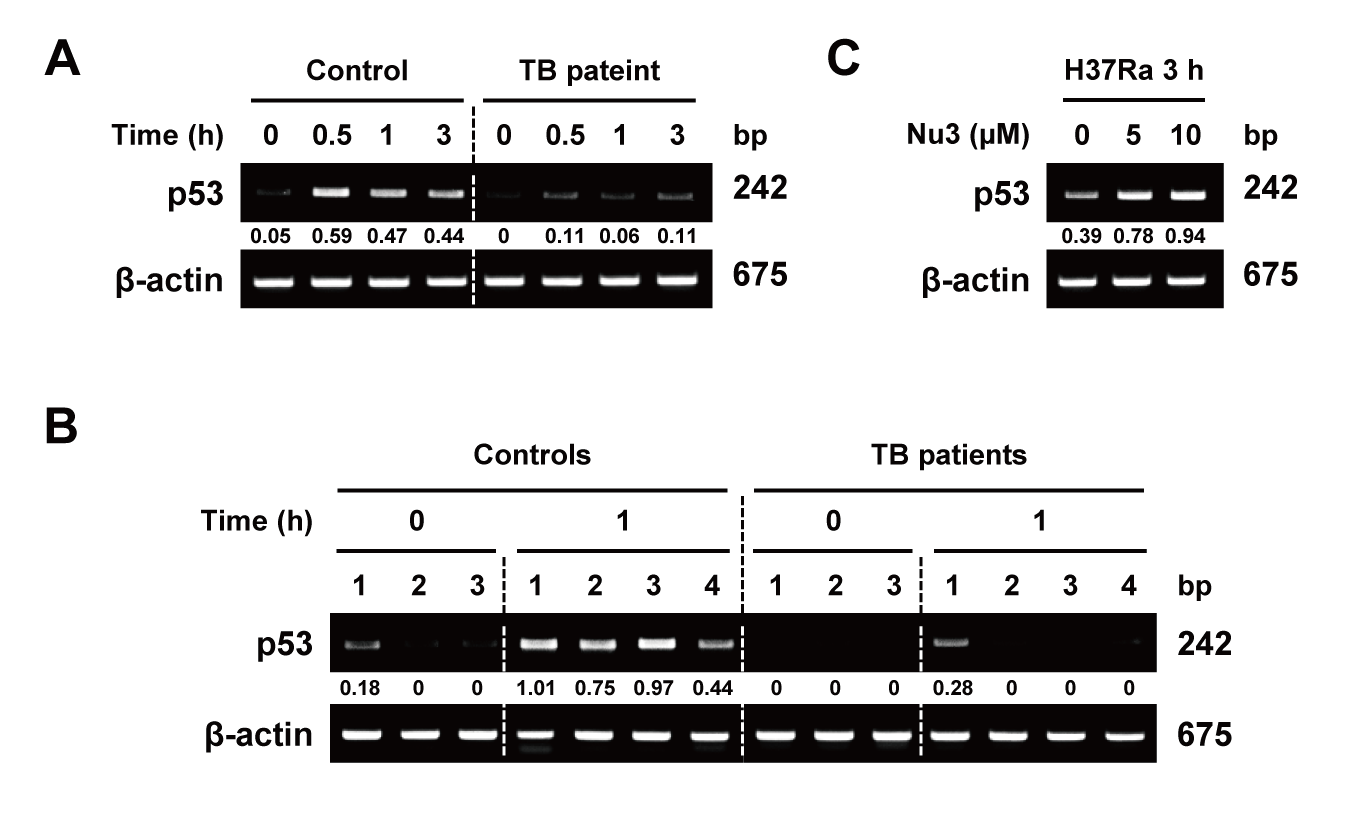


**Figure S5. p53-mediated apoptosis effectively eliminates intracellular Mtb and in the MDMs of TB patients**

(A) Blood MDMs from healthy control and TB patients were infected with H37Ra at the indicated times, and the relative expression of p53 mRNA was measured. (B) Blood MDMs of healthy controls and TB patients were infected with H37Ra for 1 h, and then p53 mRNA was detected. (D) The mRNA levels of p53 were measured in nutlin-3-treated MDMs from TB patients.
